# Linking organizational climate for evidence-based practice implementation to observed clinician behavior in patient encounters: a lagged analysis

**DOI:** 10.1186/s43058-022-00309-y

**Published:** 2022-06-11

**Authors:** Nathaniel J. Williams, Emily M. Becker-Haimes, Simone H. Schriger, Rinad S. Beidas

**Affiliations:** 1 School of Social Work, Boise State University, 1910 University Drive, Boise, ID 83625 USA; 2 Department of Psychiatry, Perelman School of Medicine, University of Pennsylvania, Philadelphia, USA; 3 Hall Mercer Community Mental Health, University of Pennsylvania Health System, Philadelphia, USA; 4 Department of Psychology, University of Pennsylvania, Philadelphia, USA; 5 Department of Medical Ethics and Health Policy, Perelman School of Medicine, University of Pennsylvania, Philadelphia, USA; 6 Department of Medicine, Perelman School of Medicine, University of Pennsylvania, Philadelphia, USA; 7 Penn Implementation Science Center at the Leonard Davis Institute of Health Economics (PISCE@LDI), University of Pennsylvania, Philadelphia, USA; 8 Penn Medicine Nudge Unit, University of Pennsylvania Health System, Philadelphia, USA; 9 Center for Health Incentives and Behavioral Economics, Perelman School of Medicine, University of Pennsylvania, Philadelphia, USA

**Keywords:** Evidence-based practice, Implementation climate, Cognitive behavioral therapy, Adherence

## Abstract

**Background:**

Theory and empirical research suggest organizational climate for evidence-based practice (EBP) implementation may be an important and malleable target to improve clinician use of EBPs in healthcare; however, this work has been criticized for overreliance on self-report measures of implementation outcomes and cross-sectional designs. This study combines data from two studies spanning 7 years to test the hypothesis that higher levels of organizational EBP implementation climate prospectively predicts improved clinician adherence to an EBP, cognitive behavioral therapy (CBT), as rated by expert observers.

**Methods:**

Biennial assessments of EBP implementation climate collected in 10 community mental health agencies in Philadelphia as part of a systemwide evaluation (time 1) were linked to subsequent observer ratings of clinician adherence to CBT in clinical encounters with 108 youth (time 2). Experts rated clinician adherence to CBT using the Therapy Process Observation Coding System which generated two primary outcomes (a) maximum CBT adherence per session (i.e., highest rated CBT intervention per session; depth of delivery) and (b) average CBT adherence per session (i.e., mean rating across all CBT interventions used; depth and breadth of delivery).

**Results:**

On average, time 2 clinician adherence observations occurred 19.8 months (*SD* = 10.15) after time 1 organizational climate assessments. Adjusting for organization, clinician, and client covariates, a one standard deviation increase in organizational EBP implementation climate at time 1 predicted a 0.63-point increase in clinicians’ maximum CBT adherence per session at time 2 (*p* = 0.000), representing a large effect size (*d* = 0.93; 95% CI = 0.63–1.24) when comparing organizations in the upper (*k* = 3) versus lower tertiles (*k* = 3) of EBP implementation climate. Higher levels of time 1 organizational EBP implementation climate also predicted higher time 2 average CBT adherence per session (*b* = 0.23, *p* < 0.001, *d* = 0.72). Length of time between assessments of climate and adherence did not moderate these relationships.

**Conclusions:**

Organizational EBP implementation climate is a promising predictor of clinicians’ subsequent observed adherence to CBT. Implementation strategies that target this antecedent may improve the delivery of EBPs in healthcare settings.

Contributions to the literature

- Theory and preliminary research suggest healthcare leaders can increase clinicians’ delivery of evidence-based practices by fostering an organizational climate that supports evidence-based practice implementation; however, this linkage has not been demonstrated in studies with independently rated observed behavior.
- This study shows that clinicians who delivered care in organizations with higher levels of evidence-based practice implementation climate demonstrated significantly higher use of evidence-based practices in clinical encounters compared to clinicians delivering care in organizations with low levels of implementation climate.
- This study offers evidence that evidence-based practice implementation climate is a promising target for implementation efforts.

## Background

Identifying levers to improve the delivery of evidence-based practices (EBPs) in healthcare is central to the field of implementation science, which aims to systematically identify how to increase the uptake of EBPs to optimize clinical outcomes [1]. One promising target for supporting the implementation of EBPs is *organizational implementation climate*, or the extent to which there is a shared perception among employees within an organization that highly adherent use of an innovation is prioritized, expected, supported, and rewarded [2]. Implementation climate is theorized to influence organizational members’ behavior in aggregate, such that employees working in organizations with high implementation climate for a specific innovation are, on average, more likely to exhibit skillful, enthusiastic, and committed use of that innovation in their work. Focusing on the implementation of EBPs in healthcare settings, Ehrhart and colleagues [3] proposed that *EBP implementation climate* describes an organizational climate in which clinicians share perceptions that they are expected, supported, and rewarded to skillfully use EBP as a routine part of patient care. Perceptions of EBP implementation climate are believed to arise as clinicians encounter competing role demands in their work and look to their organization’s policies, procedures, and practices for cues about which demands should be prioritized when trade-offs are necessary [2, 4]. If clinicians come to believe that effective use of EBP takes precedence over competing role demands, they develop shared climate perceptions that the skillful use of EBPs is expected and supported in their organization, thereby generating a high level of EBP implementation climate [5, 6]. The level of EBP implementation climate within an organization is distinct from the level of general or “molar” climate, which describes employees’ shared perceptions of the impact of the work environment on their personal well-being [4, 7, 8].

Evidence-based practice implementation climate has been theorized to be particularly critical to the successful implementation of more complex health interventions such as behavioral health EBPs which often require ongoing support (e.g., clinical supervision) to execute with high adherence [6]. Evidence-based practices in behavioral health are comprised of sequences of complex intervention techniques, delivered within and across sessions spanning weeks or months depending on each client’s unique needs and response, to promote client behavior change and symptom improvement. For example, cognitive behavioral therapy (CBT) is a leading psychosocial EBP that contains many discrete components that are often delivered in sequences that vary as a function of the specific presenting problem. Furthermore, components are often tailored to unique situations (e.g., addressing unique cognitions or behavioral contingencies) [9–11]. Considering its complexity, successful implementation of CBT is typically predicated on ongoing clinical support within an organization (e.g., clinical supervision), underscoring the interdependent nature of EBP implementation within behavioral health and the importance of clinicians’ work environment.

Emerging evidence supports the theorized importance of EBP implementation climate in behavioral health settings [3]. Studies have shown that the level of EBP implementation climate varies significantly across provider organizations [3, 12]. There is also evidence the level of EBP implementation climate predicts behavioral health clinicians’ attitudes towards EBP [13], levels of self-reported EBP use [8], and intensity of clinical supervision content related to EBP [14]. In addition, there is evidence that within-organization change in EBP implementation climate predicts within-organization change in clinicians’ self-reported EBP use over time [15].

While promising, important limitations of this research include overreliance on self-reported clinician behavior as a primary criterion variable and frequent use of cross-sectional research designs [16]. Multiple studies have shown clinician self-reports of EBP use do not always correspond to objective, expert-coded assessments [17, 18]. This may be because self-report measures are vulnerable to a range of biases, including recall, leniency, and social desirability biases [19]. Social desirability bias is particularly concerning in this line of research because over-reporting of implementation behavior may be most likely in organizations with high levels of EBP implementation climate. Cross-sectional research designs are problematic because they lack temporal precedence between the proposed antecedent and outcome, thus obscuring potential causal effects. Much research on EBP implementation climate compounds these methodological weaknesses by relying on clinician self-report of implementation outcomes within a cross-sectional design. This approach introduces an additional methodological weakness, namely, common method bias (i.e., extraneous shared variance in antecedent and outcome variables due to assessment via the same method), which can result in biased estimates of the association between variables [20]. These limitations highlight the need for studies that prospectively test the relationship between organizational EBP implementation climate (measured as clinicians’ shared perceptions) and clinicians’ subsequent observed implementation behavior during clinical encounters.

This study combines data from two separate studies conducted in the City of Philadelphia to test the relationship between EBP implementation climate, measured as part of a system-wide assessment of EBP implementation determinants [21], and subsequently observed clinician adherence to a leading behavioral health EBP (i.e., CBT), evaluated via expert ratings of audio-recorded clinical encounters collected as part of a measurement trial ((Becker-Haimes et al.: A randomized trial to identify accurate measurement methods for adherence to cognitive-behavioral therapy, forthcoming), [22]). Hypothesis 1 stated agencies’ time 1 EBP implementation climate would be positively related to clinicians’ prospectively measured (time 2) maximum CBT adherence per session (i.e., depth of CBT delivery). Hypothesis 2 stated agencies’ time 1 EBP implementation climate would be positively related to clinicians’ prospectively measured (time 2) average CBT adherence per session (i.e., breadth + depth of CBT delivery). Importantly, studying this relationship within a single system allows for greater confidence that changes in external factors (e.g., system policies) that may influence EBP use (and/or agency climate) are not conflated with measures of organizational EBP implementation climate. Should findings corroborate earlier work, results will increase our understanding of the importance of EBP implementation climate for implementation success.

## Method

### Setting

Since 2007, the Philadelphia Department of Behavioral Health and Intellectual disAbility Services (DBHIDS) has supported the implementation of EBPs for psychiatric disorders through a series of EBP training initiatives, each involving initial in-person instruction and approximately 1 year of ongoing expert consultation and support for clinicians working in behavioral health agencies within the city [13]. Since 2013, these initiatives have been led by the DBHIDS Evidence-based Practice and Innovation Center (EPIC), which coordinates technical, fiscal, and policy changes to facilitate EBP implementation by providers in the network. At the time of data collection for these studies, EPIC-led initiatives addressed multiple EBPs with a strong emphasis on CBT models including: trauma-focused CBT [22], cognitive therapy [23], dialectical behavior therapy [24], prolonged exposure [25], and parent-child interaction therapy [26]. More details about these initiatives can be found in [27]. Results of this research are reported using the STROBE guideline [28].

### Participants and procedures

#### Agencies

The study sample was formed by linking two independent research databases which contained a subset of overlapping agencies.

##### Study 1

Data for the first study (“study 1”) were collected from 2013 to 2017 in a sample of 29 youth-serving behavioral health agencies in Philadelphia. Using an observational, repeated cross-sectional design, the goal of study 1 was to examine the relationships between agency characteristics (e.g., climate) and change in clinicians’ attitudes toward, and self-reported use of, evidence-based psychotherapy techniques during 5 years of EPIC-led initiatives [22, 29]. During the study period, the EPIC-led training and policy initiatives were available to all agencies that participated in the study; however, participation in specific EPIC-led activities was not an inclusion criterion for participation in the research. Embedded within the study 1 database were biennial assessments of agencies’ EBP implementation climates. Time 1 data for the present study were drawn from these assessments.

##### Study 2

Data for the second study (“study 2”) were collected from 2016 to 2020 in an independently sampled but partially overlapping set of 27 behavioral health agencies in the greater Philadelphia region that agreed to participate in a trial evaluating methods for assessing clinicians’ adherence to CBT for youth [22]. Agencies in this study also had access to the EPIC-led EBP initiatives described above but participation in these initiatives was not an inclusion criterion for participation in the study. The study 2 database included in-session observations of clinicians’ adherence to CBT, collected on a rolling basis to accommodate patient flow and study resources (average of 2.29 observations per clinician). These assessments represent the time 2 data used in the present study.

#### Clinicians and youth

##### Study 1

Time 1 measures of EBP implementation climate were derived from confidential surveys completed by clinicians who worked with youth in agencies that participated in study 1 at linked waves. Details on clinician sampling for study 1 are provided elsewhere [21], but briefly, clinician inclusion criteria were intentionally broad to optimize the validity of the climate assessments. The overall response rate across waves was 60%. Surveys were collected directly by researchers to minimize demand characteristics and clinicians received $50 for participating. Table 1 summarizes the professional and demographic characteristics of the subsample of *N* = 90 clinicians in 10 agencies from study 1 who provided climate data used in the present study.

**Table 1:** Time 1 agency and clinician characteristics

**Agency characteristics** (*N* = 10)	*M*	*SD*
EBP implementation climate (0–4)	2.03	0.36
Molar climate (*t*-score, *μ* = 50, *σ* = 10)	63.87	7.57
**Clinician characteristics** (*N* = 90)	*M* (*n*)	*SD* (%)
Age in years	35.78	12.34
Years of clinical experience	6.93	7.86
Tenure in organization (in years)	2.65	4.53
Gender		
Man	9	10.0
Woman	77	85.6
Not reported	4	4.4
Race^a^		
African American or Black	21	23.3
American Indian or Native Alaskan	2	2.2
Asian	5	5.6
Native Hawaiian or other Pacific Islander	0	0.0
Other race	3	3.3
White	56	62.2
Not reported	5	5.6
Ethnicity		
Hispanic/Latino	5	5.6
Not Hispanic/Latino	79	87.8
Not reported	6	6.7
Employment status		
Full-time (≥ 35 h per week)	23	25.6
Part-time (< 35 h per week)	61	67.8
Not reported	6	6.7
Participated in any city-sponsored CBT training initiative?		
Yes	44	48.9
No	42	46.7
Not reported	4	4.4

^a^Totals sum to > 100% because individuals could choose multiple race identities/categories

##### Study 2

Time 2 measures of clinician adherence to CBT were derived from audiotaped recordings of psychotherapy sessions completed by clinicians serving youth in linked study 2 agencies. Beidas et al. [22] provide details on sampling of clinicians and youth for study 2, but briefly, eligible clinicians were those who planned to use at least one CBT intervention with three youth on their caseload over the next month. Prior to clinician enrollment, the study team presented on CBT interventions to ensure that clinicians understood the types of interventions that comprise this approach and clinicians were eligible to participate only if they indicated they planned to deliver CBT interventions to their clients. Study staff worked with clinicians who enrolled in the study to identify and record three sessions that met the following eligibility criteria: (1) the clinician intended to deliver at least one CBT intervention in the session, with at least 10 min of intervention content directed to the child, (2) the client was between the ages of 7 and 24, (3) the session was in English, and (4) clients under age 18 had a legal guardian who consented to their child’s participation. Only one session per client was recorded. Whenever feasible, sessions were sampled at random by the research team based on information provided by clinicians about their caseloads. In cases where clinicians provided three or fewer potentially eligible clients, all clients were approached regarding participation in the study. First-session encounters were excluded (Becker-Haimes et al.: A randomized trial to identify accurate measurement methods for adherence to cognitive-behavioral therapy, forthcoming), [22]. Table 2 presents characteristics of the *N* = 37 clinicians and *N* = 108 youth who provided in-session observations used in this study.

**Table 2:** Time 2 clinician and client characteristics

**Clinician characteristics** (*N* = 37)	*n*	%
Age in years—*M* (*SD*)	38.26	13.98
Years of clinical experience—*M* (*SD*)	8.97	7.82
Tenure in organization (in years)—*M* (*SD*)	3.14	2.80
Strength of CBT orientation (1–5)—*M* (*SD*)	3.27	0.73
Employment status		
Full-time (≥ 35 h per week)	21	56.8
Part-time (< 35 h per week)	16	43.2
Participated in any city-sponsored CBT training initiative?		
Yes	26	70.3
No	11	29.7
Gender		
Man	7	18.9
Woman	28	75.7
Not reported	2	5.4
Race^a^		
African American or Black	9	24.3
American Indian or Native Alaskan	0	0.0
Asian	2	5.4
Native Hawaiian or other Pacific Islander	0	0.0
Other race	0	0.0
White	24	64.9
Not reported	2	5.4
Ethnicity		
Hispanic/Latino	2	5.4
Not Hispanic/Latino	34	91.9
Not reported	1	2.7
**Client characteristics** (*N* = 108)	*n*	%
Category of primary diagnosis		
Autism spectrum	5	4.6
Externalizing	44	40.7
Internalizing	53	49.1
Other (bipolar/schizophrenia)	6	5.6
Presence of comorbid psychiatric diagnoses (yes)	53	49.1
Gender		
Man	52	48.1
Woman	55	50.9
Not reported	1	.9
Race^a^		
African American or Black	62	57.4
American Indian or Native Alaskan	5	4.6
Asian	5	4.6
Native Hawaiian or other Pacific Islander	2	1.9
Other race	9	8.3
White	25	23.1
Not reported	13	12.0
Ethnicity		
Hispanic/Latino	29	26.9
Not Hispanic/Latino	72	66.7
Not reported	7	6.5
Age in years—*M* (*SD*)	13.19	4.21

^a^Totals may not sum to > 100% because individuals could choose multiple race identities/categories

#### Data linkage

**Fig. 1:**
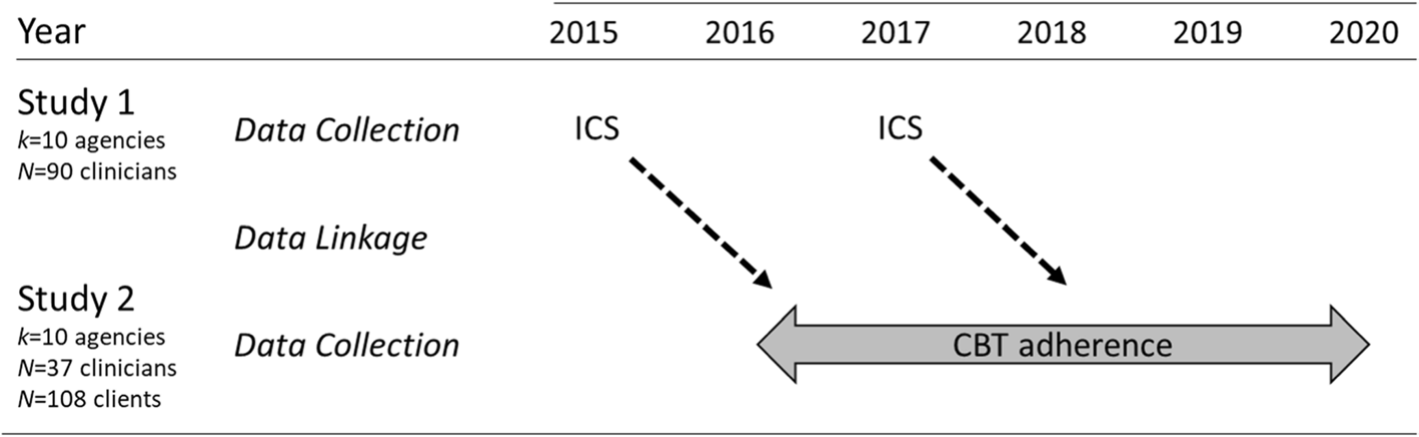
Study data collection and linkage. In study 1, agencies’ evidence-based practice implementation climate was measured in 2015 and 2017 using the Implementation Climate Scale (ICS). In study 2, clinicians’ adherence to cognitive-behavioral therapy (CBT) in sessions with youth was rated by trained coders from 2016 to 2020. Ten agencies participated in both studies which allowed linkage of the study 1 and study 2 data. The dashed arrows linking each ICS assessment to CBT adherence represents the lagged data structure in which climate assessments from study 1 were linked to subsequent CBT adherence assessments from study 2

The study dataset was formed by identifying a subset of agencies that participated in both research projects and linking their data from study 1 and study 2. To optimize the sample size, all agencies that participated in both studies and enrolled more than one clinician in study 2 were included. A total of 10 agencies met these criteria and were included in this study sample. Figure 1 shows the years of data collection and primary study variables for each project as well as the linkage structure.

The presence of biennial agency climate assessments from study 1 (spanning 2013 to 2017) overlapping with clinician session observations collected in study 2 (from 2016 to 2020) provided an opportunity to examine the lagged relationship between agency EBP implementation climate (assessed at time 1) and prospectively measured clinician adherence to CBT based on observer-coded sessions (assessed at time 2). To create this lagged structure, the two datasets were linked such that each agency’s time 1 climate assessment was the assessment from study 1 that occurred closest in time, but prior, to all of the agencies’ observations from study 2. For example, if Agency A’s climate was assessed in 2013, 2015, and 2017 as part of study 1, but clinician adherence was not assessed in Agency A until 2016, the 2015 assessment of climate was used as the “time 1” climate value for Agency A. This lagged structure maintained the temporal precedence of the hypothesized antecedent (EBP implementation climate) relative to the outcome (adherence) while minimizing the length of time between the measurement of the two variables. This is important because although climate has been shown to predict the behavior of employees working in organizations up to two years later (see [30, 31]), the goal was to minimize the time lag to provide a robust test of the study hypothesis.

### Measures

#### Clinician adherence to CBT

Clinicians’ adherence to CBT in each session was rated by trained observers who coded session audiotapes using the Therapy Process Observation Coding System-Revised Strategies (TPOCS-RS) Scale [32]. The TPOCS-RS is a widely used, gold-standard measure of CBT adherence that has demonstrated strong psychometric properties, including construct validity, internal consistency, and interrater reliability [32–34]. The scale assesses clinician adherence to 12 CBT techniques that are commonly used in sessions with youth. Observers rate the extensiveness with which clinicians deliver each of the 12 techniques in the session using a 7-point Likert scale ranging from 1 (*not present*) to 7 (*extensively*). All recorded sessions were coded by one of 11 coders, all of whom had gone through a process to establish interrater reliability. Raters attended bi-weekly meetings to prevent drift and 49% of sessions were coded by a second rater to monitor inter-rater agreement. Interrater agreement was high on all measured CBT techniques (item ICCs ranged from .76-.95). Coders were blinded to agencies’ scores on the EBP implementation climate measures from study 1.

Following prior research [34, 35], two CBT adherence outcomes were generated for each session based on observers’ TPOCS-RS ratings [32]. The two outcomes vary in the extent to which they capture depth versus breadth of CBT delivery, both of which are important for assessing adherence in a practice context [36]. *Maximum CBT adherence per session* was operationalized as the highest extensiveness rating achieved across all 12 techniques for a given session (i.e., the maximum extensiveness score for the session), ranging from 1 (*not present*) to 7 (*extensively present*). This outcome captures clinicians’ optimal performance on a single CBT technique and as such reflects depth of practice [36]. *Average CBT adherence per session* was operationalized as the average extensiveness rating (from 1 to 7) across all coded techniques within a session (i.e., mean extensiveness score of coded techniques for the session). This outcome reflects both breadth and depth of practice in a given session [36] and aligns with how this measure has been used to index CBT fidelity in prior implementation studies [34, 35].

#### Evidence-based practice implementation climate

Evidence-based practice implementation climate was measured using the well-established Implementation Climate Scale [3]. This 18-item scale includes six subscales addressing: organizational focus on EBP (*α* = .91), educational support for EBP (*α* = .86), recognition for using EBP (*α* = .86), rewards for using EBP (*α* = .87), selection of staff for EBP (*α* = .93), and selection of staff for openness (*α* = .95). Subscales are combined to produce a total score (*α* = .94). Scores on the ICS have demonstrated good evidence of reliability and validity in prior research [3, 12, 15]. In accordance with best practice guidelines for assessing organizational climate, items on the ICS incorporate a group referent (e.g., “One of this agency’s main goals…”) rather than an individual referent [6]. Items are scored on a scale from 0 (*not at all*) to 4 (*very great extent*). Consistent with theory and prior research, individual responses to the ICS were aggregated (i.e., averaged) to the agency level for analysis after confirming sufficient inter-rater agreement between clinicians within each agency using the *r*_wg(j)_ index based on a null distribution [37]. Values of *r*_wg(j)_ range from 0.0 to 1.0 with higher values indicating greater agreement. LeBreton and Senter [38] suggested values > 0.7 could be interpreted as indicating strong agreement. In this sample, all values of *r*_wg(j)_ for EBP implementation climate were > 0.7 (*M* = 0.94, *SD* = 0.04).

#### Covariates

To adjust models for potential confounds and to isolate the relationship between agency EBP implementation climate and clinician CBT adherence, agency, clinician, and client characteristics were included as covariates in all models. At the agency level, *molar organizational climate* was included, measured at time 1 using the 15-item functionality scale from the Organizational Social Context measure [39]. Molar climate represents employees’ shared perceptions of the impact of the work environment on their personal well-being [7] and generally captures the extent to which an agency is a “good” or “bad” place to work. The functionality scale has three dimensions which load onto a single factor and address clinicians’ perceptions of support and cooperation from colleagues and supervisors, role clarity within the organization, and opportunities for advancement [39]. Items are scored on a 5-point scale ranging from 1 (*never*) to 5 (*always*). Similar to EBP implementation climate, clinicians’ individual ratings on this measure are averaged to generate an agency-level variable for analysis after assessing inter-rater agreement which was excellent in this sample (mean *r*_wg(j)_ for functionality = 0.96, range *=* 0.94–0.98). Agency-level scores were converted to *t*-scores with a *μ* = 50 and *σ* = 10 based on a normative US national sample of mental health clinics [39]. Scores on the functionality scale have been linked to a range of implementation outcomes in prior research [40].

At the clinician level, the following were included: age in years, gender (reference = woman), years of clinical experience, tenure in the organization (in years), full-time vs. part-time status (> 35 h per week), strength of CBT orientation (ranging from 1 = “none” to 5 = “high”), and whether or not the clinician had participated in a post-graduate, EPIC-sponsored CBT training initiative as described above (no/yes).

At the client level, the following were included: client age in years, gender (reference = woman), primary diagnosis, and presence of comorbid diagnoses (no/yes). For analysis, client primary diagnosis was categorized as internalizing, externalizing, autism spectrum, or other (which included bipolar disorder, schizophrenic disorders, and other psychotic disorders).

### Data analytic plan

Three-level linear mixed effects regression models [41] were used to test the hypotheses that higher time 1 agency EBP implementation climate would predict time 2 clinician outcomes of (a) higher maximum CBT adherence per session and (b) higher average CBT adherence per session, while controlling for potential confounders. Separate models were estimated for each outcome. Only one session was sampled per youth; consequently, models included random intercepts at the clinician and agency levels to account for nesting of sessions within clinicians and clinicians within agencies. All models included the covariates listed above which were centered around their grand means to facilitate model interpretation and address potential differences in case mix and workforce composition across agencies [42]. Missing data on covariates were minimal (< 2%); means were imputed for missing values. To facilitate model interpretation, EBP implementation climate was standardized so that the regression coefficient represented the change in CBT adherence associated with a one standard deviation change in EBP implementation climate. Analyses were conducted in Mplus version 8 [43] using the TYPE = THREELEVEL command which employs robust maximum likelihood estimation (MLR). Each model estimated the relationship between time 1 EBP implementation climate and time 2 CBT adherence per session while holding constant all covariates.

Following model estimation, the tenability of model assumptions was checked by examining residual plots and variance inflation factor (VIF) values for all variables. All VIF values for the models were < 4, and the focal variable, EBP implementation climate, had a VIF < 2, obviating concerns regarding multicollinearity [44]. Examination of residual plots indicated there were no influential outliers or problems with heteroskedasticity or non-linearity.

Following Feingold [45], effect sizes (*d*) were calculated as the covariate-adjusted, standardized mean difference in time 2 clinician CBT adherence per session, contrasting observations from agencies in the upper versus lower tertiles of time 1 EBP implementation climate. Specifically,

$$d=\frac{M_{upper}-{M}_{lower}}{\sqrt{\upsigma_{\mathrm{outcome}}^2}}$$

where *M*_*upper*_ = the time 2 marginal mean adherence score per session in agencies in the upper tertile of time 1 EBP implementation climate, *M*_*lower*_ = the time 2 marginal mean adherence score per session in agencies in the lower tertile of time 1 EBP implementation climate, and $\sqrt{\sigma_{outcome}^2}$ = the pooled standard deviation of the time 2 CBT adherence outcome. Cohen [46] suggested standardized mean difference values of 0.2, 0.5, and 0.8 correspond to small, medium, and large effects, respectively.

## Results

Table 1 presents demographic and professional characteristics of participating clinicians who reported on agency climate at time 1. Table 2 presents characteristics of participating clinicians and clients who provided CBT adherence data at time 2. At time 2, there was significant variation across agencies in clinicians’ average age, tenure in the agency, years of clinical experience, full-time versus part-time status, self-reported strength of CBT orientation, proportion of clinicians who identified as men, and proportion of clinicians who attended city-sponsored CBT training initiatives (all *ps* < .001); all of these variables were included as covariates in the analyses. In addition, there was significant variation across agencies in the average age of clients who participated in sessions (*p* = .001), proportion of men clients (*p* = .001), and proportion of clients with primary externalizing diagnoses (*p* = .001), internalizing diagnoses (*p* = .001), and other diagnoses (psychotic or bipolar disorder, *p* = .013); these variables were also included as covariates in all analyses.

The sample mean of clinicians’ maximum CBT adherence per session was 3.57 (SD = 1.45) on a 1 to 7 scale. This corresponds to a rating in-between “*Somewhat*” and “*Considerably*” extensive adherence to the highest rated CBT technique for the session. The sample mean of clinicians’ average CBT adherence per session was 2.73 (*SD* = 0.69) on a 1 to 7 scale, corresponding to a rating of “*Somewhat*” extensive adherence to CBT across all scored techniques for the session.

On average, EBP implementation climate was measured 19.8 months (*SD* = 10.15, min = 3, max = 39) prior to measurement of clinicians’ adherence. There was no evidence of a relationship between time since measurement of EBP implementation climate and either CBT adherence outcome (all *p*s > 0.05) nor was there evidence of an interaction between climate and time since measurement of climate in predicting either adherence outcome (all *p*s > 0.5).

### Association of EBP implementation climate with maximum CBT adherence per session

Table 3 presents the parameter estimates from the linear mixed models testing the relationships between time 1 agency EBP implementation climate and clinicians’ time 2 CBT adherence per session.

**Table 3:** Models predicting clinicians’ maximum and average CBT adherence per session

	T2—maximum CBT adherence per session (*N* = 108)			T2—average CBT adherence per session (*N* = 103)		
Predictor	*B*	*SE*	*p*	*B*	*SE*	*p*
**Agency level (T1)**						
EBP implementation climate (1 SD)	0.63	0.11	0.000	0.23	0.05	0.000
Molar climate	0.00	0.02	0.927	− 0.01	0.00	0.088
**Clinician level (T2)**						
Age in years	− 0.08	0.01	0.000	− 0.03	0.01	0.000
Gender = man (ref = woman)	− 0.07	0.46	0.876	− 0.05	0.26	0.856
Years of clinical experience	0.09	0.03	0.002	0.04	0.01	0.003
Tenure in organization (in years)	− 0.08	0.06	0.204	− 0.04	0.03	0.145
Full-time employee (ref = part-time)	0.51	0.22	0.021	0.17	0.11	0.102
Strength of CBT orientation	0.31	0.10	0.003	0.15	0.05	0.003
Participated in CBT initiative (ref = no)	− 0.33	0.37	0.374	− 0.12	0.14	0.395
**Client level (T2)**						
Age in years	0.00	0.04	0.983	− 0.02	0.02	0.344
Gender = man (ref = woman)	0.38	0.35	0.283	0.19	0.18	0.279
Comorbid diagnosis (ref = no)	0.45	0.25	0.071	0.07	0.10	0.468
Primary diagnosis, ASD (ref = internalizing)	− 0.51	0.38	0.181	− 0.41	0.18	0.025
Primary diagnosis, externalizing (ref = internalizing)	0.11	0.25	0.648	− 0.11	0.10	0.301
Primary diagnosis, other (ref = internalizing)	− 0.38	0.45	0.403	− 0.33	0.22	0.143

Coefficients are estimated using 3-level, linear mixed effects regression models incorporating random intercepts for agencies (*N* = 10) and clinicians (*N* = 37). Each client was observed for only a single session. Maximum CBT adherence per session represents the highest adherence achieved (1-7) on any of 12 CBT elements for the session. Average CBT adherence per session represents the average adherence achieved across all elements scored > 1 for the session. The EBP implementation climate variable was standardized for analysis

*ASD* Autism spectrum disorder, *CBT* Cognitive behavioral therapy, *EBP* Evidence-based practice, *SD* Standard deviation

**Fig. 2:**
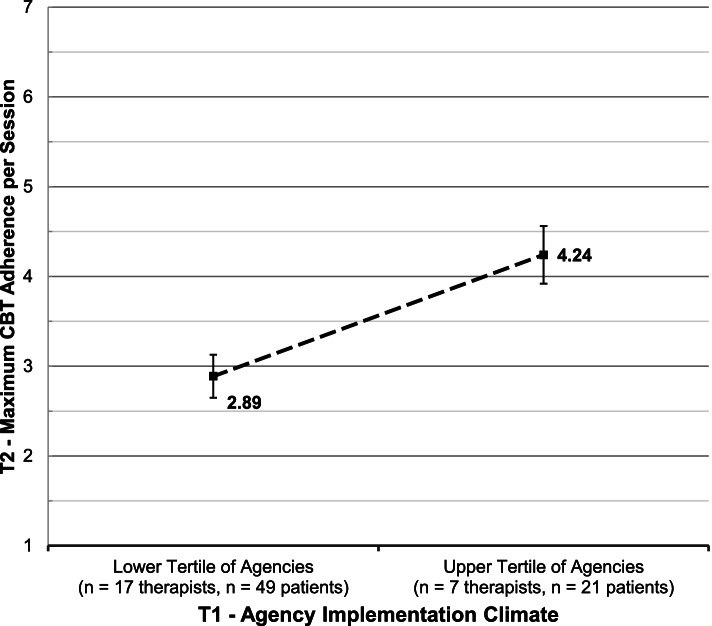
Maximum CBT adherence per session by level of agency EBP implementation climate. *N* = 10 agencies, *N* = 37 clinicians, and *N* = 108 clients. Values represent estimated time 2 marginal mean Maximum CBT adherence per session for clinicians working in agencies in the lower and upper tertiles of time 1 EBP implementation climate; error bars indicate 95% confidence intervals. Marginal means were estimated using a 3-level linear mixed effects regression model and are adjusted for all covariates. CBT, cognitive behavioral therapy; EBP, evidence-based practice; T1, time 1; T2, time 2

Consistent with hypothesis 1, higher levels of agency EBP implementation climate at time 1 were associated with higher maximum CBT adherence per session at time 2 (*b* = 0.63, *p* = 0.000). Controlling for all other variables in the model, a one standard deviation increase in time 1 EBP implementation climate was associated with a 0.63-point increase in clinicians’ time 2 maximum CBT adherence per session. This translates into a large effect size of *d* = 0.93 (95% CI = 0.63–1.24) when comparing the average, covariate-adjusted time 2 maximum CBT adherence per session in agencies with high (upper tertile) versus low (lower tertile) time 1 EBP implementation climate (see Fig. 2).

### Association of EBP implementation climate with average CBT adherence per session

**Fig. 3:**
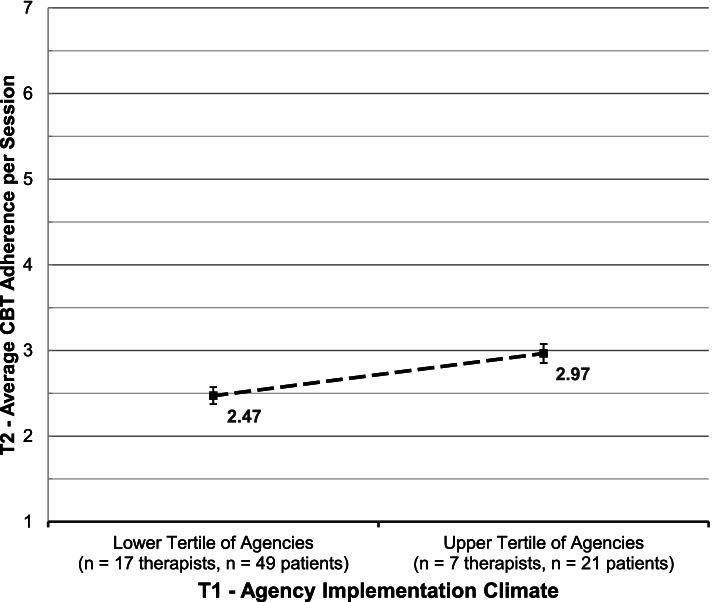
Average CBT adherence per session by level of agency EBP implementation climate. *N* = 10 agencies, *N* = 37 clinicians, and *N* = 103 clients. Values represent estimated time 2 marginal mean Average CBT adherence per session for clinicians working in agencies in the lower and upper tertiles of time 1 EBP implementation climate; error bars indicate 95% confidence intervals. Marginal means were estimated using a 3-level linear mixed effects regression model and are adjusted for all covariates. CBT, cognitive behavioral therapy; EBP, evidence-based practice; T1, time 1; T2, time 2

The results of the linear mixed models also supported hypothesis 2 which stated that time 1 EBP implementation climate would be positively related to clinicians’ time 2 average CBT adherence per session (see Table 3). Higher levels of agency EBP implementation climate at time 1 predicted significantly higher average CBT adherence per session at time 2 (*b* = 0.23, *p* = 0.000). Controlling for all other variables in the model, a one standard deviation increase in time 1 agency EBP implementation climate was associated with a 0.23-point increase in clinicians’ time 2 average CBT adherence per session. This resulted in a large effect size of *d* = 0.72 (95% CI = 0.44–1.00) when comparing the covariate-adjusted time 2 average CBT adherence per session in agencies with high versus low time 1 EBP implementation climate (see Fig. 3).

## Discussion

This study combined data from two studies spanning a total of 7 years to offer one of the first empirical tests of whether agency EBP implementation climate prospectively predicts clinicians’ observed behavior in patient encounters. Results indicated large and statistically significant associations between agency EBP implementation climate assessed at time 1 and expert-rated observed clinician behavior assessed an average of 19.8 months later (maximum CBT adherence per session *d* = .93; average CBT adherence per session *d* = .72). These results support implementation theories, models, and frameworks that hypothesize the importance of EBP implementation climate for facilitating clinicians’ use of EBP broadly and specifically within behavioral health settings. Furthermore, these findings underscore the potential value of implementation strategies that directly target EBP implementation climate in behavioral health settings and potentially beyond.

The results represent an important extension and confirmation of prior research which has linked agency EBP implementation climate to clinicians’ *self-reported* attitudes [13] and behavior [8, 12, 15] but has not yet included *objective* measures of behavior in clinical encounters. Because clinicians’ reports of implementation behavior do not always correspond to actual behavior [17, 18]; this study makes a major advance by validating the link between shared agency-level climate perceptions and clinicians’ observed implementation behavior. Furthermore, this study fills a gap in research on the time-lagged relationship between EBP implementation climate and clinicians’ prospectively measured implementation behavior by showing that EBP implementation climate measured at time 1 predicted the subsequent in-session behavior of clinicians working in the agencies an average of 19.8 months later. This is consistent with other research on organizational climate theory which suggests climate should affect the behavior of employees who remain in the organization and who join the organization over a period of up to two years [5, 30, 31].

Importantly, there was no evidence that the strength of the relationship between climate and behavior was modified by the length of time between assessments (within the observed range). This may seem surprising given that observations of CBT delivery occurred up to 39 months after the agency’s climate was assessed; however, prior research has linked climate assessments to employee and service outcomes up to two [8, 30] and three [31] years in the future. Thus, while additional research is needed, particularly in light of this study’s small sample of organizations, this finding is consistent with organizational climate theory and research which suggests climate may be relatively stable and may have lasting effects on behavior over time due to the time it takes to change organizational policies and procedures (which form the basis of shared climate perceptions), followed by the time it takes for employees to recognize and translate those changes into updated climate perceptions, and then change their behavior in response [30, 31]. As Ehrhart and colleagues noted [5]: “If climate was simply the policies, practices, and procedures of the organization, then perhaps it could be viewed as relatively easily changed . . . However, climate is not simply those things; climate is the meaning those carry as a gestalt for the organization’s employees (p. 206).” Given the typical approach most organizations take to making changes, and the amount of counter-information required to modify individuals’ images and perceptions once they are established [47], it should not be surprising that an organization’s climate may be relatively enduring over time. Longitudinal studies with large samples of organizations are needed to better understand the dynamics of change in EBP implementation climate as well as the interventions that may be most effective in generating and sustaining supportive EBP implementation climates over time.

The finding that agency EBP implementation climate predicted clinicians’ adherence to CBT, even after controlling for agency molar climate and a host of other important control variables, has important implications for the selection of implementation strategies. Taking a mechanistic perspective, the results of this study suggest EBP implementation climate may serve as an important implementation strategy target, the activation of which may improve clinicians’ implementation behavior [48]. Theory suggests organizational climate emerges from employees’ shared perceptions of their organization’s policies, procedures, and practices, with special emphasis on how these are interpreted and communicated by organizational leaders [5, 49]. Consequently, implementation strategies that target these organizational elements may support EBP implementation in behavioral health settings. Two promising strategies that may address EBP implementation climate include the Leadership and Organizational Change for Implementation strategy (LOCI) [50] and the Training in Implementation Practice Leadership (TRIPLE) strategy [51]. The LOCI strategy provides leadership training and consultation on how to modify organizational policies and practices to generate an EBP implementation climate. It has shown promise in pilot research and is currently the subject of larger trials [50, 52–54]. The TRIPLE strategy focuses on assisting agency leaders to assess the quality of services, match appropriate and feasible EBPs to the service setting, and use data to monitor quality and lead practice change. The TRIPLE strategy improved EBP implementation climate in a pre-post pilot evaluation [51]. If these strategies are found to be effective in large-scale trials, they represent a meaningful approach to supporting implementation by activating EBP implementation climate.

The results of this study raise important questions that are ripe for exploration in future research. One important set of questions pertains to the downstream association of EBP implementation climate with patient clinical outcomes via clinician CBT adherence, namely (a) what level of CBT adherence is necessary to improve patient clinical outcomes, and (b) what level of EBP implementation climate is necessary to support the targeted level of CBT adherence? In this study, CBT adherence was relatively low, even in agencies in the top tertile of EBP implementation climate (mean maximum CBT adherence per session = 4.24 and mean average CBT adherence per session = 2.97 on a 1–7 scale); however, the average level of EBP implementation climate in the top tertile was just above the mid-point of the scale (*M* = 2.39 on a 0 to 4 scale) suggesting room for improvement. Research that establishes methods for identifying clinically meaningful benchmarks for implementation outcomes would go a long way toward helping the field of implementation science understand and communicate the importance of implementation outcomes that do not have inherently meaningful metrics. Once these guidelines are established, additional studies are needed to understand what level of EBP implementation climate is necessary to achieve targeted levels of implementation outcomes. Other important questions raised by this study pertain to how EBP implementation climate interacts with clinician- and client-level factors and how these might be optimally leveraged to support EBP delivery. For example, are there individual clinician characteristics that amplify (or nullify) the effects of EBP implementation climate? How could those be optimally intervened upon to improve implementation? Sequential multiple assignment randomized trials [55, 56] could be used to generate optimized implementation strategies that address targets at multiple levels (e.g., EBP implementation climate at the agency level and clinician motivation at the clinician level) in a stepped manner [57]. These lines of inquiry represent fruitful ground for future research to guide implementation practice.

Findings should be interpreted within the context of the study limitations. As this was not a randomized experiment, causal interpretation is not indicated. Further, the extent to which these results generalize beyond this setting, behavioral health, or health care more broadly awaits further research. The agencies in this sample came from a single large urban system that was actively supporting the implementation of CBT in numerous ways. While this shared policy environment eliminates one potential confound that may “explain away” these results, it also raises the question of generalizability. For example, the supportive EBP implementation policy environment may have improved participating agencies’ EBP implementation climates in study 1 and clinicians’ CBT adherence in study 2. Future research is needed to better understand how features of the outer setting, such as system-level policies, interact with features of the inner setting, such as organizational climate, in shaping EBP implementation. Second, we relied on CBT adherence outcomes that reflect the *extensiveness* of clinicians’ CBT delivery; however, there are other ways to index CBT fidelity, such as global in-session competency [58] or a total count of the discrete CBT interventions delivered [32]. An important area for future research will be to understand which adherence outcomes are most strongly related to clinical outcomes to inform future efforts to predict fidelity in implementation research. Third, while the use of observers to code clinicians’ CBT adherence eliminated the potential for social desirability biases to inflate clinicians’ self-reported adherence ratings, it is not known if observers’ biases influenced ratings of adherence. Although there was high concordance between coders on the 49% of sessions that were double-coded in this study, future research is needed to better understand the types of biases that influence coders’ ratings of adherence. Fourth, the organizational sample size of this study is small which also raises the issue of generalizability. While these findings are consistent with other types of climate research conducted in large samples of healthcare facilities (see [59] for a review of studies on safety and quality climate in healthcare), the results nonetheless need to be replicated in future research with larger samples. Finally, although we attempted to randomly select sessions for observation, clinicians were involved in the process and may have selected sessions that reflected more extensive use of CBT. That said, there was substantial variability in observed CBT delivery and low overall observed CBT suggests this may not be a major threat in this study. Strengths of this study include its use of observer-rated clinician behavior within clinical encounters as a criterion variable, use of a gold-standard measure of EBP implementation climate and high agreement of clinicians on this measure, temporal precedence between the presumed cause (climate) and effect (CBT adherence), and inclusion of numerous rigorous statistical controls including molar organizational climate.

## Conclusions

This study fills an important gap in understanding the extent to which behavioral health agencies’ EBP implementation climate predicts clinicians’ future observed behavior in patient encounters. The results suggest there is a practically important and statistically significant relationship between agency EBP implementation climate and clinicians’ subsequent in-session adherence to CBT, above and beyond other relevant covariates. Implementation strategies that target this antecedent may improve the delivery of EBP in behavioral healthcare settings.

## Data Availability

All authors had full access to all the data in the study and take responsibility for the integrity of the data and the accuracy of the data analysis. Requests for access to deidentified data can be sent to Rinad Beidas, rinad.beidas@pennmedicine.upenn.edu.

## Abbreviations

CBT: Cognitive behavioral therapy
EBP: Evidence-based practice
ICS: Implementation Climate Scale
TPOCS-RS: Therapy Process Observation Coding System-Revised Strategies
VIF: Variance inflation factor

## References

[1] Eccles MP, Mittman BS (2006). Welcome to implementation science. Implement Sci..

[2] Klein KJ, Sorra JS (1996). The challenge of innovation implementation. Acad Manage Rev..

[3] Ehrhart MG, Aarons GA, Farahnak LR (2014). Assessing the organizational context for EBP implementation: the development and validity testing of the Implementation Climate Scale (ICS). Implement Sci..

[4] Zohar DM, Hofmann DA, Kozlowski SWJ (2012). Organizational culture and climate. The Oxford handbook of organizational psychology.

[5] Ehrhart MG, Schneider B, Macey WH (2014). Organizational climate and culture: an introduction to theory, research, and practice.

[6] Weiner BJ, Belden CM, Bergmire DM, Johnston M (2011). The meaning and measurement of implementation climate. Implement Sci..

[7] James LR, Choi CC, Ko C-HE, McNeil PK, Minton MK, Wright MA, Kim K-i (2008). Organizational and psychological climate: a review of theory and research. Eur J Work Organ.

[8] Williams NJ, Ehrhart MG, Aarons GA, Marcus SC, Beidas RS (2018). Linking molar organizational climate and strategic implementation climate to clinicians' use of evidence-based psychotherapy techniques: cross-sectional and lagged analyses from a 2-year observational study. Implement Sci..

[9] Butler AC, Chapman JE, Forman EM, Beck AT (2006). The empirical status of cognitive-behavioral therapy: a review of meta-analyses. Clin Psychol Rev..

[10] Chorpita BF, Daleiden EL (2009). Mapping evidence-based treatments for children and adolescents: application of the distillation and matching model to 615 treatments from 322 randomized trials. J Consult Clin Psychol..

[11] Wolk CB, Becker-Haimes EM, Fishman J, Affrunti NW, Mandell DS, Creed TA (2019). Variability in clinician intentions to implement specific cognitive-behavioral therapy components. BMC Psychiatry..

[12] Lyon AR, Cook CR, Brown EC, Locke J, Davis C, Ehrhart M, Aarons GA (2018). Assessing organizational implementation context in the education sector: confirmatory factor analysis of measures of implementation leadership, climate, and citizenship. Implement Sci..

[13] Powell BJ, Mandell DS, Hadley TR, Rubin RM, Evans AC, Hurford MO, Beidas RS (2017). Are general and strategic measures of organizational context and leadership associated with knowledge and attitudes toward evidence-based practices in public behavioral health settings? A cross-sectional observational study. Implement Sci.

[14] Pullmann MD, Lucid L, Harrison JP, Martin P, Deblinger E, Benjamin KS, et al. Implementation climate and time predict intensity of supervision content related to evidence based treatment. Front Public Health. 2018. 10.3389/fpubh.2018.00280. 10.3389/fpubh.2018.00280 PMC6180155 30338253

[15] Williams NJ, Wolk CB, Becker-Haimes EM, Beidas RS (2020). Testing a theory of strategic implementation leadership, implementation climate, and clinicians’ use of evidence-based practice: a 5-year panel analysis. Implement Sci..

[16] Meza RD, Triplett NS, Woodard GS, Martin P, Khairuzzaman AN, Jamora G, Dorsey S (2021). The relationship between first-level leadership and inner-context and implementation outcomes in behavioral health: a scoping review. Implement Sci..

[17] Hogue A, Dauber S, Henderson CE, Liddle HA (2014). Reliability of therapist self-report on treatment targets and focus in family-based intervention. Adm Policy Ment Health..

[18] Hurlburt MS, Garland AF, Nguyen K, Brookman-Frazee L (2010). child and family therapy process: concordance of therapist and observational perspectives. Adm Policy Ment Health..

[19] Nederhof AJ (1985). Methods of coping with social desirability bias: a review. Eur J Soc Psychol..

[20] Podsakoff PM, MacKenzie SB, Lee J-Y, Podsakoff NP (2003). Common method biases in behavioral research: a critical review of the literature and recommended remedies. J Appl Psychol..

[21] Beidas RS, Aarons G, Barg F, Evans A, Hadley T, Hoagwood K, Marcus S, Schoenwald S, Walsh L, Mandell DS (2013). Policy to implementation: evidence-based practice in community mental health – study protocol. Implement Sci..

[22] Beidas RS, Maclean JC, Fishman J, Dorsey S, Schoenwald SK, Mandell DS, Shea JA, McLeod BD, French MT, Hogue A, Adams DR, Lieberman A, Becker-Haimes EM, Marcus SC (2016). A randomized trial to identify accurate and cost-effective fidelity measurement methods for cognitive-behavioral therapy: project FACTS study protocol. BMC Psychiatry..

[23] Stirman SW, Buchhofer R, McLaulin JB, Evans AC, Beck AT (2009). Public-academic partnerships: the Beck Initiative: a partnership to implement cognitive therapy in a community behavioral health system. Psychiatr Serv..

[24] Linehan MM (2014). DBT Skills Training Manual.

[25] Foa EB, Hembree EA, Cahill SP, Rauch SA, Riggs DS, Feeny NC, Yadin E (2005). Randomized trial of prolonged exposure for posttraumatic stress disorder with and without cognitive restructuring: outcome at academic and community clinics. J Consult Clin Psychol..

[26] Eyberg S (1988). Parent-child interaction therapy. Behav Ther..

[27] Powell BJ, Beidas RS, Rubin RM, Stewart RE, Wolk CB, Matlin SL, Weaver S, Hurford MO, Evans AC, Hadley TR, Mandell DS (2016). Applying the policy ecology framework to Philadelphia’s behavioral health transformation efforts. Adm Policy Ment Health..

[28] Von Elm E, Altman DG, Egger M, Pocock SJ, Gøtzsche PC, Vandenbroucke JP (2007). The Strengthening the Reporting of Observational Studies in Epidemiology (STROBE) statement: guidelines for reporting observational studies. Bull of the World Health Org..

[29] Beidas RS, Williams NJ, Becker-Haimes EM, Aarons GA, Barg FK, Evans AC, Jackson K, Jones D, Hadley T, Hoagwood K, Marcus SC, Neimark G, Rubin RM, Schoenwald SK, Adams DR, Walsh LM, Zentgraf K, Mandell DS (2019). A repeated cross-sectional study of clinicians’ use of psychotherapy techniques during 5 years of a system-wide effort to implement evidence-based practices in Philadelphia. Implement Sci..

[30] Klein KJ, Conn AB, Sorra JS (2001). Implementing computerized technology: an organizational analysis. J Appl Psychol..

[31] Schneider B, White SS, Paul MC (1998). Linking service climate and customer perceptions of service quality: test of a causal model. J Appl Psychol..

[32] McLeod BD, Smith MM, Southam-Gerow MA, Weisz JR, Kendall PC (2015). Measuring treatment differentiation for implementation research: the therapy process observational coding system for child psychotherapy revised strategies scale. Psychol Assess..

[33] McLeod BD, Weisz JR (2010). The therapy process observational coding system for child psychotherapy-strategies scale. J Clin Child Adolesc Psychol..

[34] Smith MM, McLeod BD, Southam-Gerow MA, Jensen-Doss A, Kendall PC, Weisz JR (2017). Does the delivery of cbt for youth anxiety differ across research and practice settings?. Behav Ther..

[35] Beidas RS, Becker-Haimes EM, Adams DR, Skriner L, Stewart RE, Wolk CB, Buttenheim AM, Williams NJ, Inacker P, Richey E, Marcus SC (2017). Feasibility and acceptability of two incentive-based implementation strategies for mental health therapists implementing cognitive-behavioral therapy: a pilot study to inform a randomized controlled trial. Implement Sci..

[36] Garland AF, Hurlburt MS, Hawley KM (2006). Examining psychotherapy processes in a services research context. Clin Psychol Sci Prac..

[37] James LR, Demaree RG, Wolf G (1993). rwg: an assessment of within-group interrater agreement. J Appl Psychol..

[38] LeBreton JM, Senter JL (2008). Answers to 20 questions about interrater reliability and interrater agreement. Organ Res Methods..

[39] Glisson C, Landsverk J, Schoenwald S, Kelleher K, Hoagwood KE, Mayberg S, Green P (2008). Assessing the organizational social context (OSC) of mental health services: implications for research and practice. Adm Policy Ment Health..

[40] Williams NJ, Glisson C, Albers B, Shlonsky A, Mildon R (2020). Changing organizational social context to support evidence-based practice implementation: a conceptual and empirical review. Implementation Science 3.0.

[41] Raudenbush SW, Bryk AS (2002). Hierarchical linear models: applications and data analysis methods.

[42] Hofmann DA, Gavin MB (1998). Centering decisions in hierarchical linear models: implications for research in organizations. J Manag Organ..

[43] Muthén LKM, Muthén BO. Statistical analysis with latent variables. 8th ed. Los Angeles: Muthén & Muthén; 2017.

[44] Craney TA, Surles JG (2002). Model-dependent variance inflation factor cutoff values. Qual Eng..

[45] Feingold A (2009). Effect sizes for growth-modeling analysis for controlled clinical trials in the same metric as for classical analysis. Psychol Methods..

[46] Cohen J. Statistical power analysis for the behavioral sciences. 2nd ed. New York: Lawrence Erlbaum Associates; 1988.

[47] Lord RG, Hanges PJ (1987). A control systems model of organizational motivation: theoretical development and applied implications. Behavioral Sci..

[48] Raghavan R, Munson MR, Le C (2019). Toward an experimental therapeutics approach in human services research. Psychiatr Serv..

[49] Birken SA, Lee SY, Weiner BJ, Chin MH, Schaefer CT (2013). Improving the effectiveness of health care innovation implementation: middle managers as change agents. Med Care Res Rev..

[50] Aarons GA, Ehrhart MG, Farahnak LR, Hurlburt MS (2015). Leadership and organizational change for implementation (LOCI): a randomized mixed method pilot study of a leadership and organization development intervention for evidence-based practice implementation. Implement Sci..

[51] Proctor E, Ramsey AT, Brown MT, Malone S, Hooley C, McKay V (2019). Training in implementation practice leadership (TRIPLE): evaluation of a novel practice change strategy in behavioral health organizations. Implement Sci..

[52] Aarons GA, Ehrhart MG, Moullin JC, Torres EM, Green AE (2017). Testing the leadership and organizational change for implementation (LOCI) intervention in substance abuse treatment: a cluster randomized trial study protocol. Implement Sci..

[53] Brookman-Frazee L, Stahmer AC (2018). Effectiveness of a multi-level implementation strategy for ASD interventions: study protocol for two linked cluster randomized trials. Implement Sci..

[54] Egeland KM, Skar A-MS, Endsjø M, Laukvik EH, Bækkelund H, Babaii A, Granly LB, Husebø GK, Borge RH, Ehrhart MG, Sklar M, Brown CH, Aarons GA (2019). Testing the leadership and organizational change for implementation (LOCI) intervention in Norwegian mental health clinics: a stepped-wedge cluster randomized design study protocol. Implement Sci..

[55] Almirall D, Compton SN, Gunlicks-Stoessel M, Duan N, Murphy SA (2012). Designing a pilot sequential multiple assignment randomized trial for developing an adaptive treatment strategy. Stat Med..

[56] Lei H, Nahum-Shani I, Lynch K, Oslin D, Murphy SA (2012). A “SMART” design for building individualized treatment sequences. Annu Rev Clin Psychol..

[57] Kilbourne AM, Almirall D, Eisenberg D, Waxmonsky J, Goodrich DE, Fortney JC, Kirchner JE, Solberg LI, Main D, Bauer MS, Kyle J, Murphy SA, Nord KM, Thomas MR (2014). Protocol: Adaptive implementation of effective programs trial (ADEPT): cluster randomized SMART trial comparing a standard versus enhanced implementation strategy to improve outcomes of a mood disorders program. Implement Sci..

[58] Goldberg SB, Baldwin SA, Merced K, Caperton DD, Imel ZE, Atkins DC, Creed T (2020). The structure of competence: evaluating the factor structure of the Cognitive Therapy Rating Scale. Behav Ther..

[59] West MA, Topakas A, Dawson JF. Climate and culture for health care. In: Schneider B, Barbera KM, editors. The Oxford handbook of organizational climate and culture: Oxford University Press; 2014. p. 335–59.

